# Isolation and functional analysis of peridroplet mitochondria from murine brown adipose tissue

**DOI:** 10.1016/j.xpro.2020.100243

**Published:** 2020-12-30

**Authors:** Jennifer Ngo, Ilan Y. Benador, Alexandra J. Brownstein, Laurent Vergnes, Michaela Veliova, Michael Shum, Rebeca Acín-Pérez, Karen Reue, Orian S. Shirihai, Marc Liesa

**Affiliations:** 1 Department of Medicine, Division of Endocrinology and Department of Molecular and Medical Pharmacology, David Geffen School of Medicine at UCLA, Los Angeles, CA 90095, USA; 2 Department of Chemistry and Biochemistry at UCLA, Los Angeles, CA 90095, USA; 3 Metabolism Theme, David Geffen School of Medicine at UCLA, Los Angeles, CA 90095, USA; 4 Department of Human Genetics, David Geffen School of Medicine at UCLA, Los Angeles, CA 90024, USA; 5 Molecular Biology Institute at UCLA, Los Angeles, CA 90095, USA

**Keywords:** Metabolism, Molecular biology

## Abstract

Mitochondria play a central role in lipid metabolism and can bind to lipid droplets. However, the role and functional specialization of the population of peridroplet mitochondria (PDMs) remain unclear, as methods to isolate functional PDMs were not developed until recently. Here, we describe an approach to isolate intact PDMs from murine brown adipose tissue based on their adherence to lipid droplets. PDMs isolated using our approach can be used to study their specialized function by respirometry.

For complete information on the use and execution of this protocol, please refer to Benador et al. (2018).

## Highlights

• Isolation of peridroplet mitochondria (PDMs) from brown adipose tissue is described
• The function of murine PDMs is analyzed using 96-well format respirometry
• QC steps of PDM isolation by imaging and protein biochemistry are defined

## Graphical abstract

**Figure:**
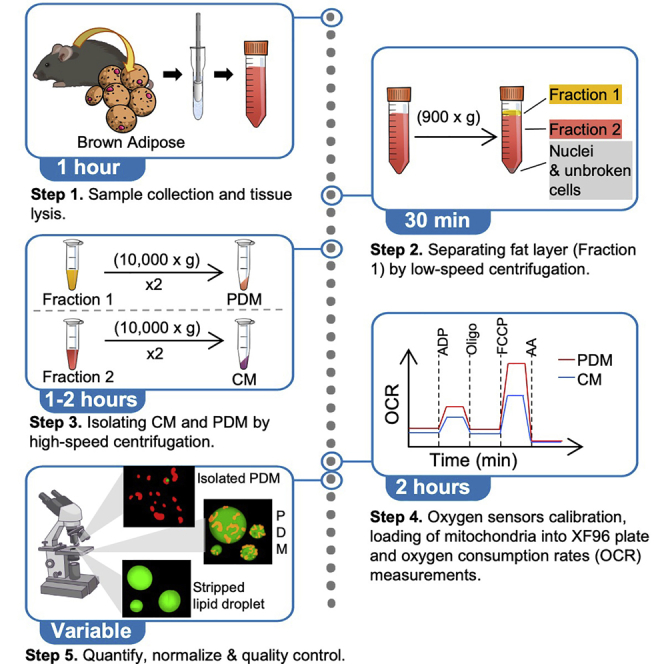

## Before you begin

**Timing: 4 h**

1. Prepare batches of the Mitochondrial Isolation Buffer (MIB), Mitochondrial Assay Solution (MAS) and respirometry substrates solutions. The use of batch preparations reduces variability across independent isolations. Storage at −20°C of these buffers in single-use aliquots for 6 months is recommended, as freeze-thawing cycles can damage respirometry substrates. For MAS + GDP without substrates, single-use aliquots of 20 mL will provide sufficient buffer for the 96 wells in a plate/assay. For buffers containing 10× respirometry substrates with and without ADP, single-use aliquots of 1.5 and 2 mL respectively will provide sufficient buffer for one 96-well plate/assay. Refer to Materials and equipment tables for more details about the buffers that are stored at −20°C and buffers that are prepared on the day of the assay.

**CRITICAL:** Adding respirometry substrates, such as pyruvate and malate, will acidify the MAS. Thus, the pH needs to be adjusted to pH 7.2 after adding substrates: pH ≤ 6 inhibits mitochondrial respiration.

**Figure 1:**
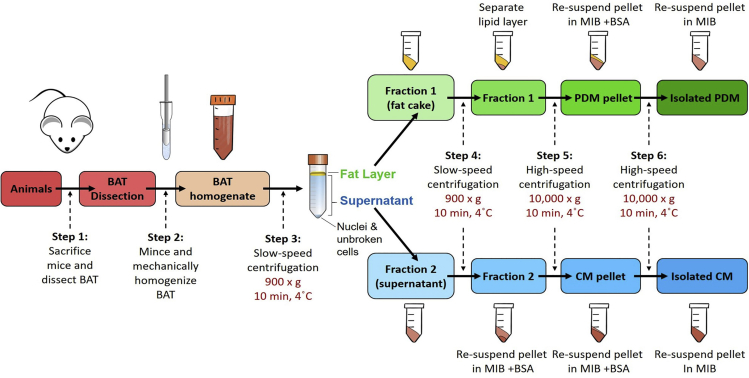
Schematic representation of peridroplet mitochondria (PDMs) isolation procedure Brown adipose tissue (BAT) is dissected from mice and homogenized with glass-Teflon Dounce homogenizer. Low-speed centrifugation generates a floating fat layer containing PDMs (fraction 1) and a supernatant containing cytoplasmic mitochondria (CMs, fraction 2). High-speed centrifugation strips and pellets PDMs from lipid droplets (LDs) and pellets CM mitochondria from the supernatant. Note that some traditional BAT mitochondrial isolation protocols discard the fat layer and/or begin with a high-speed centrifugation step.

2. MIB stocks should be prepared with and without BSA. BSA is needed to trap free fatty acids released during adipose tissue homogenization, which can uncouple and damage mitochondria. However, BSA will interfere with the protein quantification assay needed to determine the yield of isolated mitochondria. Thus, the buffer without BSA is only used for the final resuspension of the mitochondrial pellet (Figure 1).

**CRITICAL:** All solutions, materials, and equipment should be pre-cooled to 4°C (or ice-cold) before isolation and remain ice-cold throughout the protocol. These low and non-freezing temperatures preserve the integrity of mitochondria during the isolation procedure.

3. The cartridge containing the oxygen sensors from Agilent Seahorse must be hydrated for at least 4 h before the assay. It is recommended that the template and protocol for the respirometry assay in the XF96 Analyzer software be created before isolation. The respirometry protocol is as follows:

**Table:** 

Steps	Time	Cycles
**Calibration**		

Mix	2 min 0 s	2
Delay	2 min 0 s	
Mix	0 min 24 s	1
Measure	4 min 0 s	
Mix	0 min 48 s	

**Injection, port A (ADP)**		

Mix	0 min 40 s	1
Measure	4 min 0 s	
Mix	0 min 20 s	1
Measure	4 min 0 s	
Mix	0 min 48 s	

**Injection, port B (oligomycin)**		

Mix	0 min 30 s	2
Delay	2 min 0 s	
Measure	4 min 0 s	
Mix	0 min 48 s	1

**Injection, port C (FCCP)**		

Mix	0 min 24 s	2
Measure	4 min 0 s	
Mix	0 min 48 s	1

**Injection, port D (antimycin A)**		

Mix	0 min 24 s	4
Measure	4 min 0 s	

## Key resources table

**Table:** 

REAGENT or RESOURCE	SOURCE	IDENTIFIER
**Biological samples**		

Interscapular brown adipose tissue from 12 week-old C57BL/6J mice	Jackson Laboratory	Cat#000664

**Chemicals, peptides, and recombinant proteins**		

Fatty acid-free bovine serum albumin	EMD Millipore	Cat#126575
BODIPY 493/503 (4,4-difluoro-1,3,5,7,8-pentamethyl-4-bora-3a,4a-diaza-s-indacene)	Thermo Fisher Scientific	Cat#D3922
MitoTracker deep red FM	Thermo Fisher Scientific	Cat#M22426
MitoTracker green FM	Thermo Fisher Scientific	Cat#M7514
GDP (guanosine 50-diphosphate sodium type I)	Sigma-Aldrich	Cat#G7127
Pyruvic acid	Fisher	Cat#PB356
L-(−)-Malic acid	Sigma-Aldrich	Cat#M6413
Ultrapure dimethyl sulfoxide (DMSO)	Amresco	Cat#N182
KCl	Sigma-Aldrich	Cat#31248
KH_2_PO_4_	Sigma-Aldrich	Cat#P5655
MgCl_2_	Sigma-Aldrich	Cat#M0250
Ultrapure water	Invitrogen	Cat#10977-015
Succinic acid	Sigma-Aldrich	Cat#S9512
Rotenone	Sigma-Aldrich	Cat#R8875
Palmitoyl-L-carnitine chloride	Sigma-Aldrich	Cat#P1645
ADP (adenosine 5′-diphosphate monopotassium)	Sigma-Aldrich	Cat#A5285
Oligomycin A	Calbiochem	Cat#495455
FCCP	ENZO	Cat#BML-CM120-0010
Antimycin A	ENZO	Cat#ALX380075M010
PBS	Sigma	Cat#P3813
Sucrose	Fisher	Cat#L-12686
HEPES	Fisher	Cat#M-12211
EGTA	Sigma	Cat#E3889

**Critical commercial assays**		

Pierce BCA	Thermo Fisher Scientific	Cat#23225

**Software and algorithms**		

ImageJ	N/A	https://imagej.net/
Seahorse Wave Software	Agilent	www.agilent.com/en/product/cell-analysis/real-time-cell-metabolic-analysis/xf-software/seahorse-wave-desktop-software-740897nline
Excel, Prism, or spreadsheet software	Microsoft, GraphPad	https://www.graphpad.com/scientific-software/prism/

**Other**		

Glass/smooth Teflon Dounce homogenizer 10 mL	Thomas	Cat#3431D76
50 mL conical tubes	Nunc/Falcon	N/A
2 mL microfuge tubes	Eppendorf	N/A
1.5 mL microfuge tubes	Eppendorf	N/A
Surgical scissors	Fine Science Tools	Cat#91401-12
Graefe forceps	Fine Science Tools	Cat#11150-10
Fine surgical scissors	Fine Science Tools	Cat#91460-11
Noyes scissors	Fine Science Tools	Cat#15011-12
6-well plate or petri dishes	N/A	N/A
Ice buckets	N/A	N/A
Syringe and 23G needle (for exsanguination)	BD Precision Glide	Cat#305143
Sorvall ST 16R	Thermo Scientific	75004241
TX-200 swinging bucket rotor and multi-well plate adaptor	Thermo Scientific	75003658
Microcentrifuge	Eppendorf	5417C
1 mm glass slide	Fisher Scientific	Cat#22-034-985
#1.5 thickness coverslips	Zeiss	Cat#10474379
Confocal fluorescence microscope	Zeiss	LSM880
XF96 Extracellular Flux Analyzer	Agilent	XF96 Analyzer

## Materials and equipment

**Table:** Mitochondrial isolation buffer (MIB)

Reagent	Stock concentration	Final concentration	Volume/weight
Sucrose	n/a	250 mM	0.855 g
HEPES	n/a	5 mM	11.91 mg
EGTA	n/a	2 mM	7.60 mg
ddH_2_O	n/a	n/a	10 mL
**Total**	**n/a**	n/a	**10 mL**

Stir at 20°C–25°C until dissolved; bring to pH 7.2 using KOH or HCl, as Na^+^ can affect mitochondrial function. Store at −20°C for 6 months, single-use aliquots of 1 mL (<1 mL used per group of 6 mice).

**Table:** Mitochondrial isolation buffer (MIB) + BSA

Reagent	Stock concentration	Final concentration	Volume/weight
Sucrose	n/a	250 mM	17.11 g
HEPES	n/a	5 mM	238.31 mg
EGTA	n/a	2 mM	152.14 mg
BSA	n/a	2% (w/v)	2 mg/200 mL
ddH_2_O	n/a	n/a	200 mL
**Total**	**n/a**	n/a	**200 mL**

**CRITICAL:** Use fatty acid-free BSA and pH with KOH, as Na^+^ can affect mitochondrial function. Store at −20°C for 6 months in single-use aliquots of 20 mL, with a total of 7–17 mL being used per group of 6 mice, depending on tissue weight.

**Table:** Mitochondria assay solution (MAS)

Reagent	Stock concentration	Final concentration	Volume/weight
KCl	n/a	155 mM	2.143 g
KH_2_PO_4_	n/a	10 mM	340.225 mg
MgCl_2_	n/a	2 mM	101.65 mg
HEPES	n/a	5 mM	297.875 mg
EGTA	n/a	1 mM	95.09 mg
BSA	n/a	0.1% (w/v)	0.25 g
ddH_2_O	n/a	n/a	250 mL
**Total**	**n/a**	n/a	**250 mL**

**CRITICAL:** Use fatty acid-free BSA and pH using KOH, as Na^+^ can affect mitochondrial function. Store at −20°C for 6 months.

**Table:** Respirometry substrate stock solutions

Reagent	Stock concentration	Solvent
Pyruvate	0.5 M, pH 7.2	MAS + GDP
Malate	0.5 M, pH 7.2	MAS + GDP
Succinate	0.5 M, pH 7.2	MAS + GDP
Rotenone	40 mM	DMSO
Palmitoyl-carnitine	10 mM	95% v/v ethanol

**CRITICAL:** Adjust pH to 7.2. Rotenone is a poisonous reagent that must be handled with gloves and disposed of in accordance with safety data provided by supplier. Store solutions for 6 months with the exception of palmitoyl-carnitine (3 months) and rotenone (1 year).

**Table:** Mitochondrial stress compounds

Reagent	Stock concentration	Solvent
Oligomycin	20 mM	DMSO
FCCP	20 mM	DMSO
Antimycin A	40 mM	DMSO

**CRITICAL:** Oligomycin, FCCP, and antimycin A are poisonous reagents that need to be handled with gloves and disposed of in accordance with safety data provided by supplier. Store at −20°C for <1 year, aliquots for 10 assays.

**Table:** MAS + GDP (UCP1 inhibitor)

Reagent	Stock concentration	Final concentration	Volume/weight
GDP	n/a	1 mM	88.6 mg
MAS	n/a	n/a	200 mL
**Total**	**n/a**	n/a	**200 mL**

Store at −20°C for 6 months in single-use aliquots of 20 mL, which will provide buffer for 96 wells (1 plate/assay).

**Table:** Complex I driven respiration (MAS + GDP + pyruvate + malate)

Reagent	Stock concentration	Final concentration	Volume/weight
Pyruvate	0.5 M	50 mM	2 mL
Malate	0.5 M	50 mM	2 mL
MAS + GDP buffer	n/a	n/a	16 mL
**Total**	**n/a**	n/a	**20 mL**

This is a 10× solution that is loaded directly into the wells of a XF96 plate, together with mitochondria in MAS + GDP. Store at −20°C for 6 months in single-use aliquots of 2 mL, which will provide buffer for 96 wells (1 plate/assay).

**Table:** Complex I driven State 3 respiration (MAS + GDP + pyruvate + malate + ADP)

Reagent	Stock concentration	Final concentration	Volume/weight
ADP	n/a	35 mM	263.16 mg
MAS + GDP + PM buffer	n/a	n/a	15 mL
**Total**	**n/a**	n/a	**15 mL**

This is a 10× solution that is loaded into port A of the XF96 cartridge to induce maximal ATP synthesis (State 3 respiration). Store at −20°C for 6 months in single-use aliquots of 1.5 mL, which will provide buffer for 96 wells (1 plate/assay, 15 μL × 96 wells = 1.44 mL).

**Table:** Complex II driven respiration (MAS + GDP + succinate + rotenone)

Reagent	Stock concentration	Final concentration	Volume/weight
Succinate	0.5 M	50 mM	2 mL
Rotenone	40 mM	20 μM	10 μL
MAS + GDP buffer	n/a	n/a	18 mL
**Total**	**n/a**	n/a	**20 mL**

**CRITICAL:** Rotenone is a poisonous reagent that must be handled with gloves and disposed of in accordance with safety data provided by supplier. This is a 10× solution loaded into the wells of a XF96 plate, together with mitochondria in MAS + GDP. Store at −20°C for 6 months in single-use aliquots of 2 mL, which will provide buffer for 96 wells (1 plate/assay).

**Table:** Complex II driven respiration (MAS + GDP + succinate + rotenone + ADP)

Reagent	Stock concentration	Final concentration	Volume/weight
ADP	n/a	35 mM	263.16 mg
MAS + GDP + SR buffer	n/a	n/a	15 mL
**Total**	**n/a**	n/a	**15 mL**

This is a 10× solution that is loaded into port A of the XF96 cartridge to induce maximal ATP synthesis (State 3). Store at −20°C for 6 months in single-use aliquots of 1.5 mL, which will provide buffer for 96 wells (1 plate/assay, 15 μL × 96 wells = 1.44 mL).

**Table:** Fatty acid-fueled respiration (MAS + GDP + palmitoyl-carnitine)

Reagent	Stock concentration	Final concentration	Volume/weight
Palmitoyl-carnitine	10 mM	40 μM	80 μL
Malate	0.5 M	1 mM	40 μL
MAS + GDP buffer	n/a	n/a	20 mL
**Total**	**n/a**	n/a	**20 mL**

This is a 10× solution that is loaded into the Seahorse well, together with mitochondria in MAS + GDP. Store at −20°C for 3 months in single-use aliquots of 2 mL, which will provide buffer for 96 wells (1 plate/assay).

**Table:** Fatty acid-fueled State 3 respiration (MAS + GDP + palmitoyl-carnitine + ADP)

Reagent	Stock concentration	Final concentration	Volume/weight
ADP	n/a	35 mM	263.16 mg
MAS + GDP + PC buffer	n/a	n/a	15 mL
**Total**	**n/a**	n/a	**15 mL**

This is a 10× solution loaded into port A of the XF96 cartridge to induce maximal ATP synthesis (State 3). Store at −20°C for 6 months in single-use aliquots of 1.5 mL, which will provide buffer for 96 wells (1 plate/assay, 15 μL × 96 wells = 1.44 mL).

**Table:** State 4o respiration (MAS + GDP + substrate + oligomycin)

Reagent	Stock concentration	Final concentration	Volume/weight
Oligomycin	20 mM	40 μM	4 μL
MAS + GDP + substrate	n/a	n/a	2 mL
**Total**	**n/a**	n/a	**2 mL**

Add oligomycin (poisonous) to MAS + GDP on the day of the assay. This is a 10× solution that is loaded into port B of the XF96 cartridge to block ATP synthesis (16.6 μL × 96 wells = 1.59 mL).

**Table:** Maximal respiration (MAS + GDP + substrate + FCCP)

Reagent	Stock concentration	Final concentration	Volume/weight
FCCP	20 mM	40 μM	4 μL
MAS + GDP + substrate	n/a	n/a	2 mL
**Total**	**n/a**	n/a	**2 mL**

Add FCCP (poisonous) to MAS + GDP on the day of the assay. This is a 10× solution that is loaded into port C of the XF96 cartridge to induce maximal respiration (18.5 μL × 96 wells = 1.78 mL).

**Table:** MAS + GDP + substrate + antimycin A

Reagent	Stock concentration	Final concentration	Volume/weight
Antimycin A	40 mM	40 μM	2 μL
MAS + GDP + substrate	n/a	n/a	2 mL
**Total**	**n/a**	n/a	**2 mL**

Add antimycin A (poisonous) to MAS + GDP on the day of the assay. This is a 10× solution that is loaded into port D of the XF96 cartridge to block mitochondrial respiration (20.5 μL × 96 wells = 1.97 mL).

**Table:** Mitochondria and lipid droplet imaging dye stock solutions

Reagent	Stock concentration	Solvent
BODIPY 493/503	1 mM	DMSO
MitoTracker green FM	1 mM	DMSO
MitoTracker deep red FM	1 mM	DMSO

Store at −20°C in aliquots of 10μL for 6 months.

**Table:** Imaging solution A

Reagent	Stock concentration	Volume/weight
BODIPY 493/503	1 mM	1 μL
MitoTracker red	1 mM	1 μL
MAS + GDP + PM buffer	n/a	1 mL
**Total**	**n/a**	**1 mL**

**Table:** Imaging solution B

Reagent	Stock concentration	Volume/weight
MitoTracker Green	1 mM	1 μL
MitoTracker red	1 mM	1 μL
MAS + GDP + PM buffer	n/a	1 mL
**Total**	**n/a**	**1 mL**

**CRITICAL:** Imaging solution A and B should be prepared freshly on the day of the assay.

## Step-by-step method details

This mitochondrial isolation protocol uses the Agilent Seahorse XF96 Analyzer to analyze respiratory function. Note that oxygen consumption can be analyzed in these isolated mitochondrial fractions using other equipment as well. The primary adjustment that might be required when using other equipment is to increase the amount of mitochondrial mass loaded per measurement.

### Sample collection

**Timing: 30 min**

The first section of this protocol describes the dissection and preparation of murine brown adipose tissue, prior to its mechanical lysis to liberate mitochondria and lipid droplets. We are currently adapting this protocol to other mouse and human tissues, which will be described in a separate publication.

**Figure 2:**
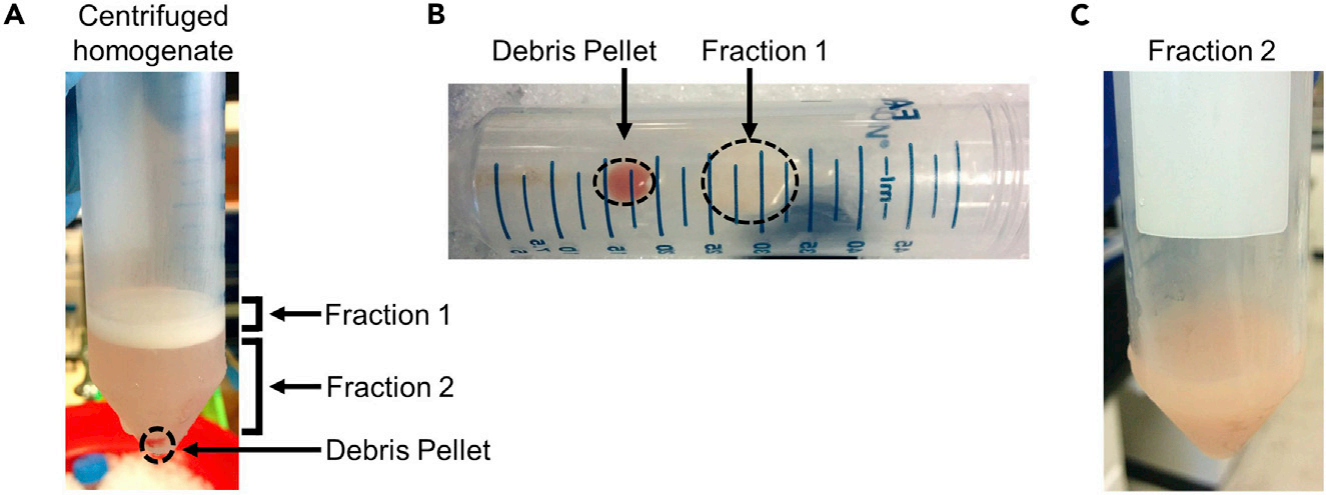
Separation of the fat layer containing PDMs by low-speed centrifugation (A) Image of 50 mL Falcon tube containing BAT homogenate after first low-speed centrifugation (step 3). Note the separation of fraction 1, containing the fat layer and peridroplet mitochondria (PDMs), from fraction 2, containing cytosolic mitochondria (CMs). (B and C) (B) Fraction 1 in the decanted original Falcon tube after pouring (C) the supernatant containing CMs, namely fraction 2, into a clean tube.

***Note:*** The number of mice needed for effective isolation of PDMs varies according to tissue lipid content. A sufficient amount of lipids is required to effectively form a buoyant fat layer on the surface of the homogenate (see Figure 2). Fewer animals can be used if mice have higher lipid content due to age, diet, or genetic manipulation. This protocol has been optimized for BAT isolation from 6 mice, with dissected BAT weights per mouse varying between 80–300 mg. Thus, a minimum of 480 mg of tissue is needed.

**CRITICAL:** The buoyancy of lipid droplets is the process that separates peridroplet mitochondria (PDMs) from the other mitochondria.

1. Euthanize mice one-by-one using isoflurane and secondary cervical dislocation as approved by your institutional animal care and use committee in compliance with US Public Health Service Regulation. Cervical dislocation should be performed carefully to avoid tearing interscapular brown adipose tissue at the base of the neck.

**CRITICAL:** Isoflurane must be handled in a fume hood, as it is a volatile anesthetic.

2. Exsanguinate mice by cardiac puncture using a syringe with a 23G needle to minimize blood within the isolated BAT.
3. Dissect interscapular BAT from 6 male 12-week-old C57BL/6J mice and immediately place in 6-well plate or petri dish containing ice-cold PBS.
4. Use surgical scissors and forceps to separate BAT from connective tissue, muscle, and white adipose tissue, preferably using a dissection microscope.
5. Weigh pooled BAT tissue from 6 mice (480 mg minimum) and mince it into ∼2mm pieces with scissors or razors.
6. Re-suspend tissue pieces in 10:1 (volume: weight) in ice-cold MIB + BSA.

### Tissue and cell mechanical lysis

**Timing: 10 min**

**CRITICAL:** All tubes and materials must be ice-cold and remain ice-cold throughout the protocol.

7. Disrupt tissue with 9–10 strokes in ice-cold glass/Teflon Dounce homogenizer until the MIB and tissue appear as a homogenous solution, with the Dounce head moving smoothly through the solution.

**CRITICAL:** Do not use detergents or soaps to clean the homogenizer, as they can disrupt mitochondrial membranes. Use warm water and clean brushes. For samples with higher content of connective tissue, a glass-glass loose Dounce homogenizer is recommended. As glass can break during homogenization, one should be mindful of sharps hazards.

### Separating fat cake by low-speed centrifugation

**Timing: 20 min**

Peridroplet mitochondria (PDMs) are separated from cytosolic mitochondria (CMs) during the low-speed centrifugation step, which also separates nuclei, insoluble cell debris and unbroken tissue (Figure 2A).

8. Transfer homogenate into ice-cold 50mL Falcon tube (Corning) and centrifuge 900 × *g* (low speed) for 10 min at 4°C, using a centrifuge with swinging bucket rotor.

**CRITICAL:** When using a swinging bucket rotor, measure the distance between the center of the rotor and the center of the liquid homogenate mass within the tube, when in horizontal position, in order to transform 900 × *g* into the actual rpm needed.

$$\text{rcf }\left(\text{g}\right)\;=1.12\times \text{ radius }\left(\text{mm}\right)\times {\left(\frac{\text{rpm}}{1\text{,}000}\right)}^{2}$$

9. Carefully pour the supernatant, which is fraction 2 that contains CMs, into a new ice-cold falcon tube (Figure 2C). A cell strainer (70–100 μm) can be used to prevent debris or loose fat to mix with the CM fraction. Pouring will leave the fat layer on the side of the tube, which is fraction 1 that contains PDMs, and the pellet in the original tube (Figure 2B).
10. By keeping the tube horizontally, scrape fraction 1 into a second and clean ice-cold 50 mL tube and re-suspend in 4 mL of MIB + BSA buffer (Figure 2B).

***Alternatives:*** Use a P1000 micropipette with 1/3 of its tip cut, collect fraction 1, and transfer it to a clean tube. Cutting 1/3 of the tip increases its bore size, which prevents tip clogging when aspirating the fat layer. Collecting fat layer by pipetting is not the preferred option, as fat can remain attached to the tip and thus lost. Consequently, this alternative should only be used if the fat layer does not remain intact and adherent to the side of the tube, after pouring fraction 2 to a clean tube. See Troubleshooting, problem 1.

**CRITICAL:** It is critical that sufficient tissue is homogenized to generate a compact and floating fat layer on the surface of the homogenate. See Troubleshooting, problem 1 if a floating layer is absent.

**CRITICAL:** Save 1 μL of fraction 1 for peridroplet mitochondria imaging, as a quality control assessment.

11. Discard the original 50 mL tube that contains the pelleted unbroken cells, nuclei, and debris.
12. Repeat the low-speed centrifugation on fraction 1 re-suspended in 4 mL of MIB + BSA and on fraction 2 in the new tube, to further eliminate debris.

**CRITICAL:** Fractions 1 and 2 must be completely cleared of debris prior to high-speed centrifugation steps. If necessary, perform a third low-speed centrifugation. If a residual lipid layer is generated after a second slow spin of fraction 2, discard it and do not pool it with clean fraction 1.

### Isolating CMs and PDMs by high-speed centrifugation

**Timing: 1 h 30 min**

By using high-speed centrifugation, PDMs will be separated from fraction 1 and cytosolic mitochondria (CMs) from fraction 2.

**Figure 3:**
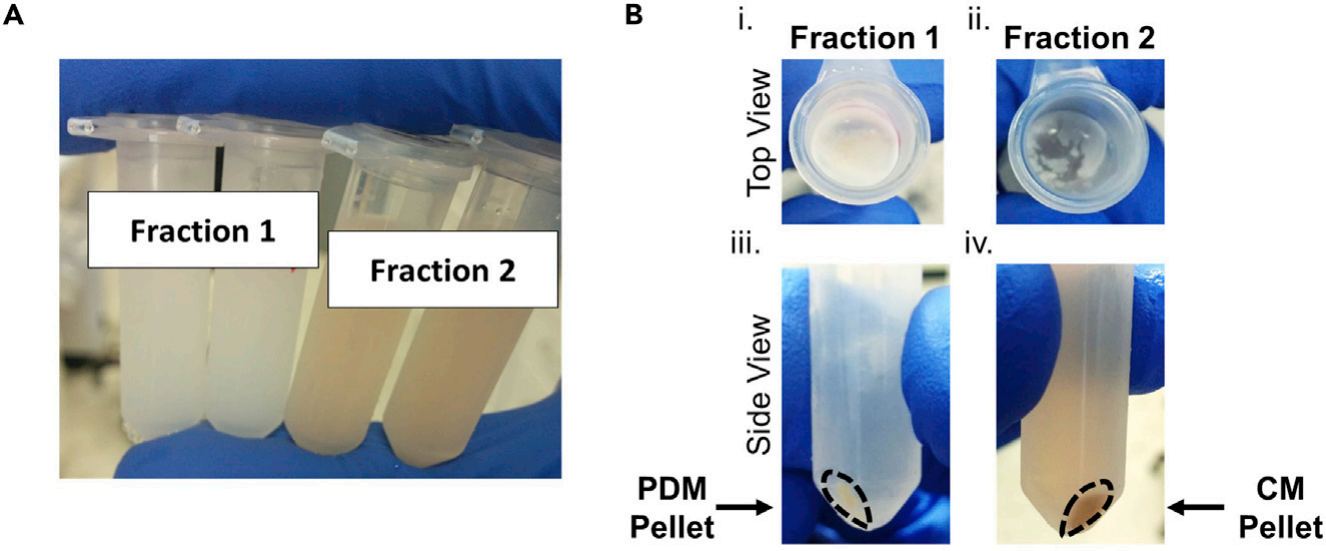
Mitochondrial isolation by high-speed centrifugation (A) Re-suspended fraction 1 in MIB + BSA and fraction 2 before high-speed centrifugation. (B) Top and side views of the mitochondrial pellets generated from high-speed centrifugation of fraction 1 and fraction 2. Note that fraction 2 after high-speed centrifugation should have minimal fat layer compared to fraction 1.

13. Transfer clean fraction 1 and fraction 2 to 2mL Eppendorf tubes and centrifuge at 10,000 × *g* for 10 min at 4°C using a microfuge (Figure 3A).

14. Discard the lipid layer and supernatants generated after centrifuging Fractions 1 and 2.
  a. Use a P1000 pipette to physically scrape the lipid layer and gently evacuate residual lipids before removing supernatant. This step is to prevent the lipids from mixing with the PDM pellet.
    **CRITICAL:** Save at least 1 μL of the discarded lipid layer for quality control measurements.
  b. The pellet obtained from fraction 1 will contain PDMs and the pellet from fraction 2 will contain CMs (Figure 3B).
  c. Re-suspend pellets in 200 μL MIB + BSA and transfer them to clean 1.5 mL tubes.
    **CRITICAL:** There should be minimal lipid content floating after high-speed centrifugation of fraction 2. The presence of large amounts of lipid in fraction 2 can indicate a contamination of CMs with PDMs. Refer to “Separating Fat Cake by Low-Speed Centrifugation” steps to optimize slow centrifugation steps and minimize lipid content in fraction 2.

15. Repeat high-speed centrifugation step to wash residual lipids and non-mitochondrial contents with MIB + BSA.
16. Following the second high-speed centrifugation, remove supernatant and preserve washed pellets with mitochondria.

**CRITICAL:** There should be no visible lipid layer at this step. Perform additional centrifugation steps until no lipid layer is observed.

17. Gently re-suspend mitochondrial pellets by pipetting up and down (do not use vortex) in ice-cold MIB without BSA and determine protein concentration by BCA assay (Thermo). When re-suspending pellet in MIB, aim for a concentration of ∼10 μg/μL (1:2–1:3 volume pellet: buffer). These ratios result in re-suspending fraction 1 in 20–40 μL and fraction 2 in 40–60 μL of buffer.

**CRITICAL:** Use MIB without BSA for protein determination, as BSA will cause an overestimation of protein content in the mitochondrial fractions. See Troubleshooting, problems 1 and 2.

**Pause point:** Isolated CMs and PDMs can be pelleted and stored long-term at −80°C. Frozen and thawed mitochondria can be used to measure maximal respiratory capacity, with the exception of mitochondrial coupled respiration (Acin-Perez et al., 2020, Osto et al., 2020). It is recommended to determine the protein concentration before use.

### Functional assessment of PDMs and CMs by respirometry

**Timing: 1 h**

Here we describe how to measure oxidative function of PDMs and CMs under different fuels, using the Seahorse XF96 analyzer from Agilent Technologies. This equipment allows the use of minimal amounts of biological sample, namely 2–4 μg of protein per measurement. Respiration can be measured in these same fractions using other equipment measuring oxygen consumption, but the amount of mitochondria loaded will likely need to be increased. Consult “Before You Begin” section to prepare the equipment and XF96 cartridge before starting the assay.

18. Load the cartridge with 15 μL MAS + ADP in port A, 16.6 μL MAS + oligomycin in port B, 18.5 μL MAS + FCCP in port C, and 20.5 μL MAS + antimycin A in Port D (see Tables with Solution composition in Materials and Methods section).

***Alternatives:*** As basal respiration with substrates in the absence of ADP is functionally equivalent to respiration under oligomycin, one can plate mitochondria already with ADP, then add 15 μL MAS + oligomycin in port A, 16.6 μL MAS + FCCP in port B, 18.5 μL MAS + antimycin A in port C and 20.5 μL MAS + TMPD/Ascorbate in Port D to determine maximal complex IV activity. This alternative is critical for models with less healthy or less resilient mitochondria. Starting at state 3 reduces the time of the assay itself and of mitochondria being hyperpolarized. The reason is that hyperpolarized, highly respiring mitochondria are not as stable at 37°C. See Troubleshooting, problem 3.

19. Load the protocol prepared the day before the assay, press start, and place the utility plate containing the hydrated sensor cartridge, with its ports loaded, into the XF96 analyzer to begin oxygen and pH sensor calibration. Calibration takes 30 min, during which one can load mitochondria into the XF96 cell culture microplate.
20. For each mitochondrial fuel, generate a master mix with sufficient volume for at least triplicate measures (3 wells), with up to three different fuels assessed per sample. Each well will contain 2–4 μg mitochondria in 20 μL MAS + the substrate to be assessed (pyruvate + malate, succinate + rotenone or palmitoyl-carnitine + malate). Thus, each master mix will contain at least a total of 60 μL with 6–12 μg of mitochondria per substrate to cover triplicates.
21. Load 20 μL of each master mix into individual wells of the microplate using a P20 pipette. Mitochondria show higher respiratory rates under succinate + rotenone. Thus, 2 μg of mitochondria should be loaded under succinate + rotenone and 4 μg for the other substrates.
  a. Centrifuge the plate at 2,000 × *g* for 5 min at 4°C using a swinging bucket rotor with plate carriers and running with the rotor brakes off, to sediment mitochondria at the bottom surface of the well.

**CRITICAL:** Load mitochondria in the center of the well. If mitochondria attach to the wall of the well, oxygen consumption will not be properly measured. Turn OFF centrifuge brake and allow the rotor to reduce speed gradually, so that the plate is not shook by a sudden decrease in speed and mitochondria can stay at the center of the well.

22. Add 115 μL MAS + GDP + substrate using multichannel pipette at a 45° angle to the top of each chamber well. Total volume in the well is going to be 135 μL. The total volume needs to be accurate, as 10× solutions for ADP (15 + 135 = 150 μL), oligomycin (16.6 + 150 = 166.6 μL), FCCP (18.5 + 166.6 = 185.1 μL), and antimycin A (20.5 + 185.1 = 205.6 μL) will be injected from the ports on top.

**CRITICAL:** The additional 115 μL of MAS should be added gradually, to prevent detaching mitochondria from the bottom of the well. Mitochondria can be visualized by bright field or fluorescent microscopy, using a 40×–63× objective. A homogenous layer of bacteria-like structures (mitochondria) should be observed on the surface of the well (see Figure 5B).

23. Warm the Seahorse plate with mitochondria in a 37°C incubator for 5 min before loading the plate into the XF96 Analyzer.
24. Replace the utility plate with the Seahorse plate loaded with mitochondria and start the assay. Assay duration is around 1 h.

### Data analysis

**Timing: 1 h**

25. Export Point-to-point oxygen consumption rates (OCRs) of individual wells from Seahorse instrument (Figure 4).

**Figure 4:**
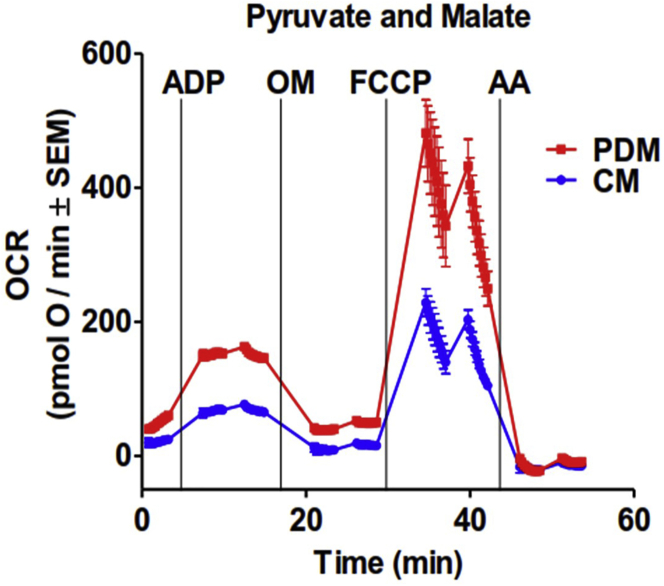
Respirometry assay of isolated PDMs versus CMs from brown adipose tissue Representative trace of oxygen consumption rates (OCR) of isolated PDMs and CMs fueled by pyruvate + malate. ADP, oligomycin, FCCP, and antimycin A were sequentially injected to assess mitochondrial respiratory states. The injection of ADP in port A increases, oligomycin decreases, and FCCP increases respiration only when mitochondria are intact after isolation. Antimycin A blocks respiration, leaving some oxygen consumption attributed to other mitochondrial processes (i.e., ROS generation). 4–6 technical replicates per group. Data are expressed as mean ± SEM. Adapted from Benador et al. (2018).
26. Check that the oxygen tension traces are linear in each measurement, as a quality control parameter. Non-linear traces do not allow a reliable calculation of OCR.
27. Using Microsoft Excel, perform the following calculations described with the OCR values measured in each individual well. For each calculation, the OCR under Antimycin A is subtracted from other values to obtain the OCR that corresponds to mitochondrial respiratory function. This is because Antimycin-resistant oxygen consumption cannot arise from mitochondrial respiration, since Antimycin A blocks electron transfer from complex III to complex IV, with complex IV being responsible for respiration (reducing oxygen to water).
  a. State 2: Subtract minimal OCR value following antimycin A injection from maximal OCR value at the start of the assay. State 2 represents respiration of mitochondria with fuels, without ATP synthesis, as ADP should be absent.
  b. State 3: Subtract minimal OCR value following antimycin A injection from maximal OCR value following ADP injection. See Troubleshooting, problem 3 if poor response to ADP is observed.
  c. State 4o: Subtract minimal OCR value following antimycin A injection from minimal OCR value following oligomycin injection (mitochondria with fuels, with ATP synthase blocked, functionally equivalent to state 2).
  d. Maximal: Subtract minimal OCR value following antimycin A injection from maximal OCR value following FCCP injection. See Troubleshooting, problem 4 if OCR is unstable.

### Quality control

**Timing: variable**

To assess the quality of isolated mitochondria, Option A describes a quantitative parameter to assess integrity of mitochondrial function after isolation. Option B describes a microscopic assessment of the lipid droplets with mitochondria still attached by fluorescence microscopy and the presence of lipid droplets in the PDM fraction. Option C describes measuring the relative mitochondrial protein content in each fraction by biochemical techniques, to determine correspondence of protein loaded to the respirometry assay with mitochondria loaded. Option D describes assessing mitochondrial function by membrane potential analysis.

28. Option A: The respiratory control ratio (RCR) is the ratio between state 3/state 2 or state 3/state 4o respiration and can be used as a quantitative measure of mitochondrial inner membrane damage. Physical damage to the mitochondrial inner membrane is indicated by RCR values < 4, when respiring under pyruvate + malate, or < 2, when respiring with succinate + rotenone or palmitoyl-carnitine + malate. A similar or greater fold increase in OCR should be seen after injecting FCCP.
29. Option B: Measurement of mitochondria and lipid droplet (LD) content by fluorescence microscopy.
  a. Combine 1 μL of non-stripped fat layer (Figure 3A), 1 μL stripped fat layer and 1 μL isolated PDMs (Figure 3B) with 1 μL Imaging solution A.
  b. Place 1 μL of each sample- Imaging solution A mix on a #1.5H coverglass and cover with an additional coverglass.
  c. Place the coverglass on a confocal microscope stage and image BODIPY using 488 nm laser for excitation and collect fluorescence emission at 500–550 nm. Using a separate track, image MitoTracker with 633 nm excitation and collect emission at 650–700 nm. Use 20× lens for low-magnification imaging of LD content (Figure 5) (Benador et al., 2018).

**Figure 5:**
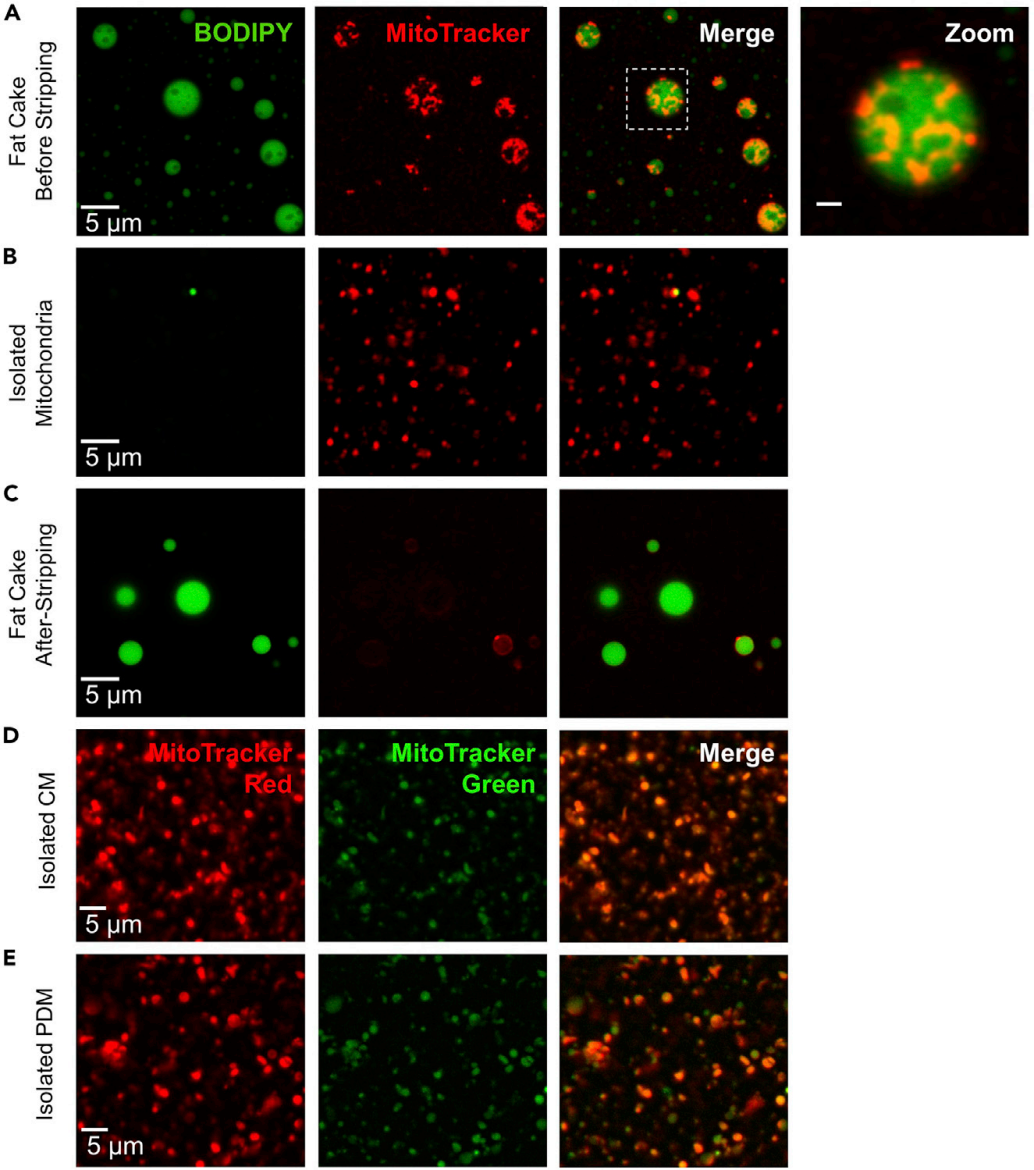
Confocal fluorescence microscopy of isolated PDMs (A) Images of re-suspended fraction 1 before high-speed centrifugation stained with the lipid droplet dye BODIPY (green) and the mitochondrial dye MitoTracker red. Note the localization of mitochondria to lipid droplet surface. (B) Image of re-suspended PDM pellet stained with mitochondrial marker MitoTracker red. Isolated PDMs have little to no lipid droplets. (C) Images of re-suspended fraction 1 after high-speed centrifugation, stained with the lipid droplet dye BODIPY (green) and the mitochondrial dye MitoTracker red. Note the lack of mitochondria. (D) Images of isolated mitochondria from fraction 2 (CMs) stained with MitoTracker green and MitoTracker red. (E) Images of isolated mitochondria from fraction 1 (PDMs) stained with MitoTracker green and MitoTracker red. Scale bars, 5μm. Adapted from Benador et al. (2018).
  d. The non-stripped fat layer should have mitochondria closely attached to lipid droplets (Figure 5A) and the isolated PDM fraction should not have lipid droplets (Figure 5B). The stripped fat layer should have little to no mitochondria (Figure 5C).
30. Option C: Quantification of total mitochondrial content in each fraction by western Blot.
  a. Combine 1–10 μg isolated mitochondria and boil at 95 °C for 5 min.
  b. Run and transfer an SDS gel as specified by gel apparatus manufacturer.
  c. Probe for the relative content of mitochondrial markers, such as TOMM20 or VDAC and quantify per microgram of protein loaded.

**CRITICAL:** The semi-quantitative data obtained from Option B can provide evidence that functional differences detected between samples of different types do not arise from differential enrichment of PDMs in the isolated fraction. Option C is an essential step when comparing PDMs from different tissues or mice with different ages or genetic manipulations.

***Alternatives:*** Evaluate mitochondrial content by mass spectroscopy proteomic analysis using MitoCarta 2.0 to estimate mitochondrial protein content.

31. Option D: Assessment of mitochondrial function by membrane potential imaging.
  a. Combine 1 μL of isolated mitochondria with 1μL Imaging solution B.
  b. Place 1 μL of mitochondria-solution B mix on a #1.5H coverglass and cover with an additional coverglass.
  c. Place the coverglass on microscope stage and image MitoTracker green using 488 nm laser excitation and 500–550 nm emission. Using a separate track, image MitoTracker red with 633 nm excitation and 650–700 nm emission (Figures 5D and 5E).

***Note:*** MitoTracker red staining is more sensitive to differences in membrane potential, while MitoTracker green is less sensitive. Therefore, mitochondria with intact membrane potential will stain with both green and red (Figures 5D and 5E), while depolarized mitochondria will only stain green (Benador et al., 2018).

## Expected outcomes

### Normal respiration patterns shared by PDMs and CMs

Basal or State 2 oxygen consumption rate (OCR) is generally between 30–60 pmol O_2_/min (Figure 4) (Benador et al., 2018) . After the injection of ADP, OCR increases at least 4-fold in mitochondria under pyruvate and malate and at least 2-fold with other substrates. Subsequent injection by oligomycin yields a drop in OCR to basal or State 2 values (see Troubleshooting, Problem 3 if this is not the case). The third injection with the mitochondrial uncoupler FCCP must induce a similar increase in OCR or higher than the increase induced by ADP. The last injection, antimycin A, blocks electron transfer from complex III to complex IV causing an immediate drop in OCR. Rates after antimycin A injection are typically under 10 pmol O_2_/min and reflects other processes that consume oxygen, but are not respiration (i.e., ROS generation). See Troubleshooting, Problem 4 if rates are unstable.

### Functional differences between CMs and PDMs

When comparing BAT CMs and PDMs, PDMs assayed using pyruvate and malate as substrate typically display a 2-fold higher OCR rates than CMs under state 3 (maximal ATP-synthesizing respiration induced by ADP injection), as well as in maximal respiration (under FCCP, Figure 4). OCR values are generally between 100–300 pmol O_2_/min for CMs and 200–600 pmol O_2_/min for PDMs. The higher values of OCR observed in PDMs compared to CMs are observed when mitochondria respire under succinate and rotenone as well (Benador et al., 2018). See Troubleshooting, Problems 2 and 4.

### Confocal images of isolated fat layer and peridroplet mitochondria

Fluorescent staining and imaging of mitochondria and LD in the fat layer will reveal numerous mitochondria attached to LDs (Figure 5A). High-speed centrifugation strips mitochondria from LDs, resulting in a pellet containing PDMs without large lipid droplets and only showing 5% of total BODIPY fluorescence (Figure 5B). Indeed, this 5% is confined to very small LD, smaller than some mitochondria. Accordingly, the stripped fat layer shows that 95% of mitochondria have been removed, with only 5% of MitoTracker red signal retained in the stripped fat layer (Figure 5C) (Benador et al., 2018).

## Limitations

Separating fraction 1 from fraction 2 can be problematic. Yield of PDMs may diminish due to an ineffective formation and buoyancy of the fat layer that constitutes fraction 1. Ineffective formation can be caused by insufficient tissue, lower total lipid content, or tissue harboring lipid droplets of very small size.

Separation of PDMs by differential centrifugation may preferentially select for PDMs attached to larger lipid droplets (LDs), which are more buoyant. Small LDs with insufficient buoyancy could potentially be lost or potentially contaminate fraction 2.

The PDM fraction might be constituted by a heterogeneous population, with PDMs behaving differently when attached to large lipid droplets, when compared to small lipid droplets. Our current approach of PDM isolation pools PDMs attached to small and to large lipid droplets together.

This protocol strips PDMs from lipid droplets by high-speed centrifugation, which means that it is a possibility that centrifugation may potentially not detach mitochondria that have a very strong interaction with lipid droplets.

Mass spectrometry analyses reveal that the crudely isolated CMs and PDMs are equally contaminated with other organelles and cellular compartments, mostly the endoplasmic reticulum (ER). Qualitative analyses reveal that 50% of the proteins detected in PDM and CM fractions are mitochondrial.

This protocol has been optimized for BAT from male C57BL/6J mice but could be extrapolated to other organisms. Optimization for other tissues is needed and we are currently developing protocols for other tissues, including liver and white adipose tissue.

## Troubleshooting

### Problem 1

Low yield of CMs and/or PDMs.

### Potential solution

Sample yields, particularly of PDMs, will differ based on the age, diet, and genetic background of the animal, as these factors determine total lipid content and lipid droplet size.

Due to the presence of multiple washes and centrifugation steps, there is high risk of losing material (mitochondria and fat). This can be minimized by increasing the amount of tissue used.

The mechanical separation of fraction 1 from fraction 2 using a plastic micropipette tip can cause fat to adhere to the tip, resulting in loss of material, including PDMs. We recommend cutting 1/3 of the P1000 tip to increase bore size and pipetting slowly to minimize sample loss in step 10.

### Problem 2

CM and PDM preparations do not yield differences in biochemical and functional assays.

### Potential solution

Due to the crude separation of fraction 1 from fraction 2, there is the potential for PDM and CM cross contamination, particularly PDM contamination of the CM fraction. The presence of a remaining lipid layer remaining in fraction 2 after high-speed centrifugation increases the likelihood of PDM-CM cross contamination. It is recommended to collect fraction 1 with more supernatant, rather than trying to get fraction 1 without any fraction 2 (supernatant) after the low-speed centrifugation.

If contamination is suspected (i.e., a fat layer is present after high-speed centrifugation of fraction 2), it will be confirmed by respirometry. PDMs have higher ATP-linked respiration and capacity to oxidize pyruvate.

PDM and CM protein concentrations may not have been accurately assessed. Since step 14 requires a MIB+BSA wash, residual BSA could remain after MIB resuspension in step 17 and will interfere with accurate protein quantification. If the amount of starting material is large, we recommend a second centrifugation of the mitochondria pellet with MIB lacking BSA. However, it should be noted that mitochondria are more prone to damage when samples are highly diluted, so we do not recommend a secondary resuspension if starting material/mitochondrial pellet is small. If normalization by total protein content is not reliable due to BSA contamination, alternative normalization could be performed: by staining the XF96 plate with MitoTracker deep red (MTDR) and measuring red fluorescence in the well or by immunoblotting. With the first approach, samples can be normalized by MTDR staining either prior or after respirometry (Acin-Perez et al., 2020). Normalization by immunoblot can be performed using TOMM20 and Porin levels.

### Problem 3

PDMs and CMs are uncoupled or show poor responses to ADP and FCCP.

### Potential solution

We recommend increasing the concentration of BSA utilized in MIB buffer and ensuring that GDP is included in the buffer. BSA is needed to trap free fatty acids released during adipose tissue homogenization, which can damage the mitochondrial inner membrane and/or activate UCP1. The addition of BSA should reduce levels of free fatty acids, and the inclusion of GDP will inhibit UCP1 and uncoupled respiration. Uncoupled PDMs could also result from physical damage incurred during homogenization. To minimize potential physical damage, ensure that the Dounce homogenizer was not washed with detergents and reduce the number of strokes to 6 during homogenization.

Another explanation is that mitochondria are not resilient to the assay. In this case, start the respirometry assay in state 3, with the mitochondria having saturating ADP concentrations when plated, to shorten the time of mitochondria being hyperpolarized (see Alternative after step 18).

### Problem 4

Oxygen consumption rates are unstable, giving a transient peak with very high values that subsequently decrease.

### Potential solution

Ensure that mitochondria are seeded at an appropriate amount per well. Optimal seeding conditions should be determined experimentally, with lower amounts required when mitochondria use succinate and rotenone. Basal oxygen consumption rates (substrates without ADP) should not exceed 250–275 pmol O_2_/min.

If high respiration rates persist after decreasing the amount of mitochondria loaded, we recommend reducing measurement times in the respirometry assay protocol commands.

## Resource availability

### Lead contact

Further information and requests for resources and reagents should be directed to and will be fulfilled by the Lead Contact, Dr. Marc Liesa (mliesa@mednet.ucla.edu).

### Materials availability

This study did not generate any unique materials or reagents.

### Data and code availability

We did not generate any unique datasets or code.
